# Century-scale changes in phytoplankton phenology in the Gulf of Maine

**DOI:** 10.7717/peerj.6735

**Published:** 2019-05-02

**Authors:** Nicholas R. Record, William M. Balch, Karen Stamieszkin

**Affiliations:** Bigelow Laboratory for Ocean Sciences, East Boothbay, ME, United States of America

**Keywords:** Phenology, Climate, Phytoplankton, Chlorophyll, Bloom, Gulf of Maine

## Abstract

The phenology of major seasonal events is an important indicator of climate. We analyzed multiple datasets of in situ chlorophyll measurements from the Gulf of Maine dating back to the early 20th century in order to detect climate-scale changes in phenology. The seasonal cycle was consistently characterized by a two-bloom pattern, with spring and autumn blooms. The timing of both spring and autumn blooms has shifted later in the year at rates ranging from ∼1 to 9 days per decade since 1960, depending on the phenology metric, and trends only emerged at time scales of >40 years. Bloom phenology had only weak correlations with major climate indices. There were stronger associations between bloom timing and physical and chemical variables. Autumn bloom initiation correlated strongly with surface temperature and salinity, and spring bloom with nutrients. A later spring bloom also correlated with an increased cohort of *Calanus finmarchicus*, suggesting broader ecosystem implications of phytoplankton phenology.

## Introduction

One of the earliest studied and most striking oceanographic phenomena is the spring phytoplankton bloom. A prominent feature of satellite images, the bloom initiates as “*flowerings of diatoms, resulting in local swarms so dense as to be the most spectacular event in the yearly planktonic cycle*” (Bigelow, 1924a). Efforts to predict the timing of the spring bloom in the Gulf of Maine helped to coalesce the interdisciplinary and quantitative approaches that underlie modern oceanography (Riley, 1949). Despite over 100 years of study and a corpus of theory, predicting the dynamics of phytoplankton blooms remains an important but elusive goal (Sverdrup, 1953; Taylor & Ferrari, 2011; Mahadevan et al., 2012; Behrenfeld & Boss, 2014; Zarubin, Lindemann & Genin, 2017).

Phytoplankton blooms are indeed spectacular events, and their timing is particularly important to marine ecosystems in strongly seasonal climes. Because they constitute the primary source of carbon fixation for the pelagic food web, animals across trophic levels have life history strategies tuned to the seasonal cycle of production. Productive systems depend on seasonal timing matches between trophic levels because mismatches in timing can mean that a grazer or predator misses its feeding window (i.e., match-mismatch hypothesis, (Cushing, 1969; Edwards & Richardson, 2004). The Gulf of Maine is a quintessential example of this phenomenon. The zooplankton community is largely copepods of the genus *Calanus* (Bigelow, 1924a), which time their emergence from diapause to exploit the spring phytoplankton bloom. The pulse of *Calanus* production is then succeeded by fish (Golet et al., 2015), mammal (Pershing et al., 2009), and other migrations.

There is growing attention to changing phenology as both an indicator and a consequence of climate change. Understanding phenology in a climate context requires time series that span many decades. Such datasets in the pelagic ocean are limited. In the Gulf of Maine, there have been noted phenological shifts in recent times, particularly regarding physical properties such as temperature (Thomas et al., 2017), stratification, and hydrology (Smith et al., 2012), related to climate change. To understand the associated phenological changes in the pelagic ecosystem in a climate context, we need biological time series that span many decades—in seasonal oceans a minimum of 40 years (Henson et al., 2010). To examine changes in phytoplankton phenology in the Gulf of Maine at the climate scale, we aggregated multiple datasets of in situ chlorophyll measurements over the past century. We used these data to address the following questions: (1) Has the timing of phytoplankton blooms changed over the past century? (2) What are the drivers of these changes? And (3) What are the ecological consequences of these changes?

## Data and Methods

### Phytoplankton dataset

**Figure 1:**
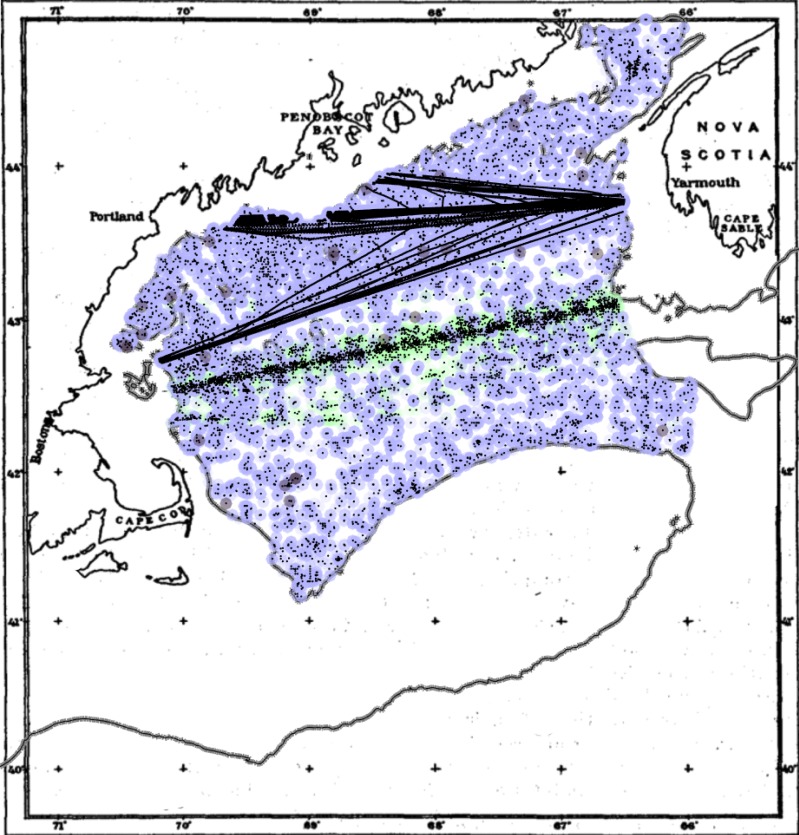
Sample locations for the compiled phytoplankton dataset in the inner Gulf of Maine. Points are back-colored by original measurement type: FU (grey), PCI (green), chlorophyll (blue). Background map adapted from (Bigelow, 1924a). The grey line is updated 100 m isobath.

We aggregated datasets of in situ phytoplankton measurements from multiple public sources. We used chlorophyll (generally chlorophyll-a) as an estimate of phytoplankton biomass and used previously published datasets. Chlorophyll time series in the interior Gulf of Maine vary together based on empirical orthogonal function analysis, separately from the coastal Gulf of Maine (Thomas, Townsend & Weatherbee, 2003). This analysis thus focused on the inner Gulf of Maine, including all samples deeper than the 100 m isobath (106,257 samples, Fig. 1). For some data, measurements were in standard units (mg chl m^−3^). For others, a conversion was necessary. For phytoplankton colour index (PCI) measured by the continuous plankton recorder (CPR) and for Forel-Ule (FU) scale, we used established relationships for conversions, which for PCI was categorical (Raitsos et al., 2005) and for FU was exponential (Wernand, Van der Woerd & Gieskes, 2013). These conversion relationships are from global datasets, contain uncertainty, and possibly unknown variability in space and time. The fact that our analysis focuses on the timing of events and is less reliant strictly on magnitude should minimize potential biases. The data were all from calibrated, quality-controlled, public databases: Aggregated database of (Boyce, Lewis & Worm, 2012, 1934–2010, mg chl m^−3^ and FU), Continuous Plankton Recorder (1961–2013, PCI), Gulf of Maine North Atlantic Time Series (1998–2015, mg chl m^−3^ and FU (Balch et al., 2016), including measurements from (Bigelow, 1924b), and the World Ocean Database (Boyer et al., 2013) (1934–2010, mg chl m^−3^ and FU). In the post-1960 period, most datasets were largely gap-free during the indicated sampling years, with the exception of FU measurements, which were sporadic throughout the time series (<1% of measurements). We averaged these data into a monthly time series, removing duplicates, spanning the time period 1912–2015.

We tested the accuracy and limitations of a temporal cubic spline interpolation for missing data. We validated the interpolation by removing each data point, interpolating, comparing the interpolated value to the measured value, and comparing the phenology metric (described below) calculated from the interpolation to that calculated from the measured value. For an interpolation that only included points not surrounded by missing data, the interpolated values compared well to measured values (*r* = 0.37, *p* ≪ 0.0001 over all points, and *r* > 0.99, *p* ≪ 0.0001 for phenology metrics). When the interpolation was tested for points surrounded by missing data, there was no significant correlation between interpolated and measured values, so we did not interpolate beyond one point.

### Oceanographic datasets

We assembled oceanographic time series to compare to chlorophyll phenology. We used previously published data for water column physics (Fisheries and Oceans Canada, climate database, http://www.bio.gc.ca/science/data-donnees/base/run-courir-en.php, (Gregory, 2004) and nutrients (Rebuck & Townsend, 2014). We used measurements that fell within the spatial domain matching the chlorophyll dataset and averaged to monthly values. Physical data included temperature (T) and salinity (S) averaged over the upper 50 m. We also included a stratification index (Δ), estimated as the difference of mean density (*σ*_*T*_) between the surface (0–50 m) and deep (50 m - bottom) layers (Drinkwater & Gilbert, 2004). Chemical data included surface NO_3_+NO_2_, Si(OH)_4_, and PO_4_ measurements, using only those measurements that passed all quality control criteria (Rebuck & Townsend, 2014). We also used annual climate indices: the Atlantic Multidecadal Oscillation (AMO), North Atlantic Oscillation (NAO), Gulf Stream Index (GSI), the Arctic Ocean Oscillation (AOO), and the Arctic Oscillation (AO) (cf NOAA Climate Prediction Center).

### Grazer dataset

We used copepod count data from the CPR. The dominant seasonal copepod in the central Gulf of Maine is *Calanus finmarchicus* (Runge et al., 2014), which has a seasonal peak in surface waters following the spring bloom. Adults emerge from diapause and exploit the spring bloom for egg production. This cohort reaches adulthood in the late spring and summer. We calculated a mean log anomaly for late-stage (copepodite 5–6) *C. finmarchicus* for May–July, as an index of how well this species fares following the spring bloom. We did not compute an analogous autumn time series because *C. finmarchicus* has entered diapause at depth by that time, and the CPR makes surface measurements.

### Analysis

We used a clustering analysis to examine the shape(s) of the bloom cycle across years. This helped to confirm that our phenology metrics were applicable, and also to detect changes in the bloom pattern across time. We clustered years based on the shape of the seasonal cycle of chlorophyll following the methodology of Foukal & Thomas (2014): briefly, we used k-means multivariate clustering, with the squared Euclidean distance as the similarity metric and randomly selected starting centroids. Other similarity metrics yielded qualitatively similar results. To account for the randomness in the algorithm, we computed an ensemble of 100 runs, sorted clusters by frequency, and analyzed the modal clusters for each year.

We produced time series of phenology metrics for spring and autumn blooms. There are many metrics for quantifying the timing of phytoplankton blooms, with most metrics targeting the timing of either bloom initiation or bloom peak (Ji et al., 2010; Brody, Lozier & Dunne, 2013). Bloom shape can vary from year to year and by location, so no single metric has emerged as the preferred choice. Because of the coarse temporal resolution (1 month), some phenology metrics were not appropriate for this dataset. We computed five phenology metrics for the spring and autumn blooms:

1. *μ*_*m*_ - Timing of maximum. Because the dataset is binned monthly, this is a coarse time series; values are midpoints of months.
2. *μ*_*c*_ - Center of mass. This metric computes the center of mass within the February-May time period (spring index) and of the August-November time period (autumn index), and provides a smooth metric.
3. *μ*_*g*_ - Midpoint of Gaussian fit. This metric fits a Gaussian curve to half of the year (for spring and autumn indices) and uses the fitted mean (µ) parameter as the bloom peak timing, providing a smooth metric.
4. *τ*_*d*_ - Timing of maximum increase. This is a coarse (monthly) index of the timing of bloom initiation.
5. *τ*_*t*_ - Threshold timing. This metric computes the timing at which the total value crosses a threshold that is some percentage of the year’s total (i.e., the cumulative sum method). We computed this metric for multiple thresholds for both spring and autumn indices.

We used the phenology metrics in time series analysis. We examined the time series for statistically significant trends. We then looked for long-term associations with physical drivers and with upper trophic levels to determine the potential causes and consequences of changing phenology. Analysis was performed using Pearson correlation and coherence analysis. The coarse spatio-temporal resolution of the dataset means that there is additional variance around time series and associated relationships, and correlation coefficients reflect that. We took steps to avoid over-reliance on single *p*-value determinations and dichotomous thinking (McShane & Gal, 2017). While we did use *p* < 0.05 as one benchmark, we disregarded those correlations driven by an outlier. Furthermore, only correlations that held up for multiple phenology metrics or with very strong relationships were considered robust. For the analysis with the climate indices and the *C. finmarchicus* time series, the time series were sufficiently long and gap-free to conduct a coherence analysis. We used the minimum variance distortionless response approach (Benesty, Chen & Huang, 2005). We also tested the climate indices at lags of 0–3 years, and for the *C. finmarchicus* time series, because of the ontogenetic lag and east-to-west hydrographic flow, we compared it to the phenology time series calculated for just the eastern Gulf of Maine (east of >68^∘^W).

## Results

### Clustering

**Figure 2:**
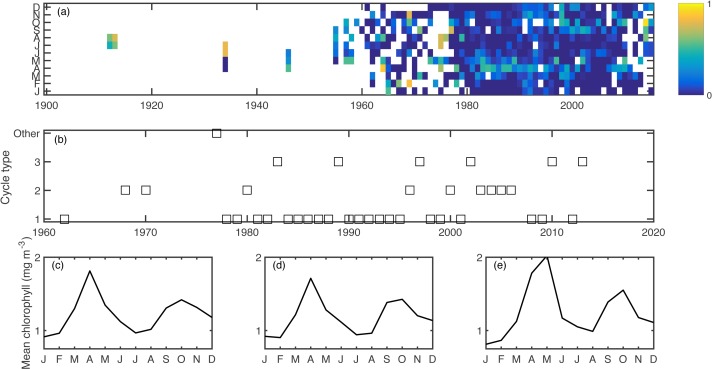
Aggregated chlorophyll dataset (A), showing monthly binned chlorophyll (log mg m^−3^), including a 1-unit spline interpolation. Years clustered by cycle type, as computed in the k-means clustering algorithm. (B) Cycle type for each year for which there are sufficient data, showing the most common three cycle types. Seasonal shapes for cycle types 1–3 (C–E) respectively, calculated as the climatology over all years within each cluster.

Excluding singletons, there were three main bloom types that emerged consistently over the ensemble of cluster calculations (Fig. 2). The most common was the conventional cycle, with a pronounced spring bloom peaking in April, a smaller autumn bloom peaking in October, and a seasonal minimum in January. This cycle type dominated in most of the 1980s and 1990s. The second most common was a two-bloom cycle where the spring and autumn blooms were of closer magnitude to each other, again peaking in April and October. The third cycle type had a pronounced but delayed spring bloom, peaking in May, a diminished autumn bloom, and a higher winter minimum. The second and third cycle patterns appeared sporadically over the later part of the time series. All of these cycle patterns had a predominantly two-peak shape, conducive to the phenology metrics used.

### Trends

**Figure 3:**
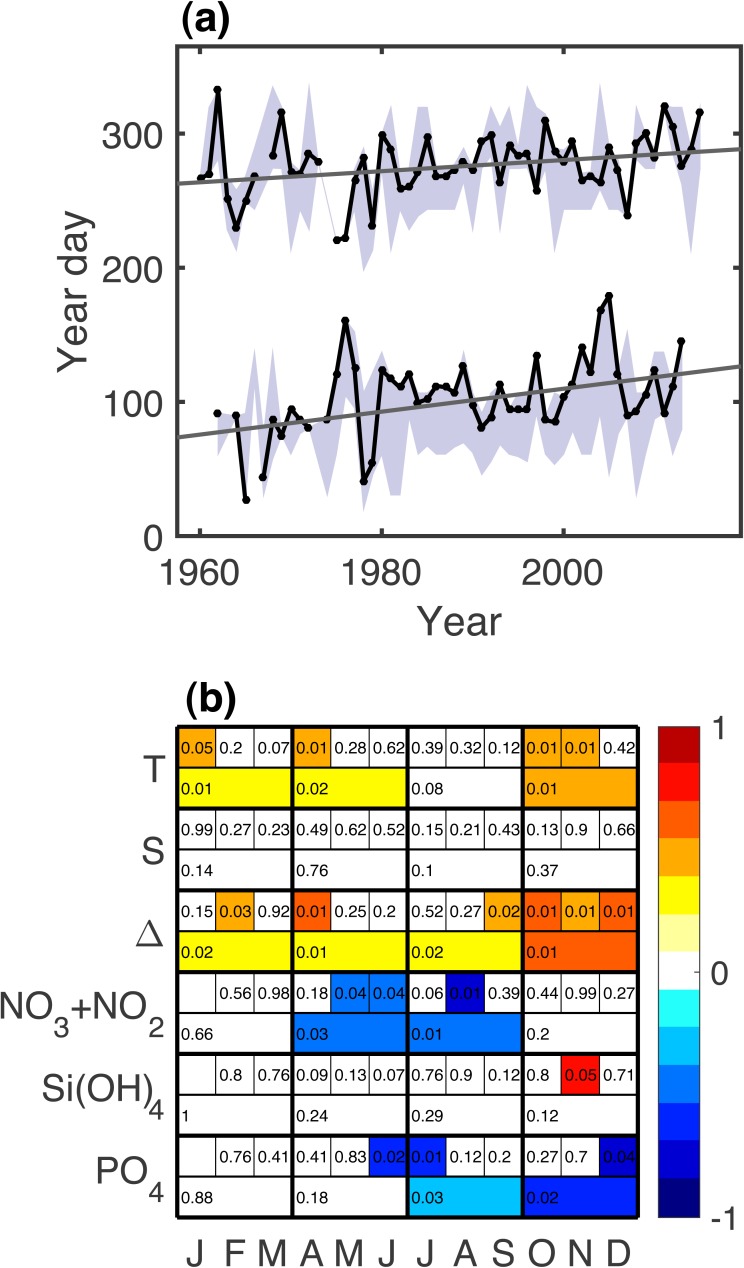
(A) Time series of spring and autumn *μ*_*g*_ and least squares line showing statistically significant increase since 1960. Shaded area shows the range of all phenology metrics. (B) Significant secular trends for the measured physical and chemical variables during different months and seasons. Color scale is the strength of the trend as measured by a correlation coefficient (*r*). Numbers are *p* values rounded up to the nearest hundredth. Trends with *p* < 0.05 are colored.

For all phenology metrics, spring and autumn, the trend over the time series was positive–i.e., shifting later in the year. The strongest trend was for *μ*_*g*_, shifting later with mean rates of 8.9 (*p* = 0.001) and 4.3 (*p* = 0.02) days per decade (spring and autumn, respectively). Other metrics with noteworthy trends were spring *μ*_*m*_ (4.4 days per decade, *p* = 0.1), spring *μ*_*c*_ (1.5 days per decade, *p* = 0.1), autumn *μ*_*c*_ (1.2 days per decade, *p* = 0.07), and spring *τ*_*t*_ (4.1 days per decade, *p* = 0.09). Taking the average of metrics, trends were significant for both spring (*p* = 0.03) and autumn (*p* = 0.01), with rates of 3.7 and 3.8 days per decade, respectively. Over the past sixty-year period, where there are enough data to consistently capture the phenology metrics, these shifts equate to a roughly 10–50 day shift in timing. There is substantial inter-annual variance around these trends (Fig. 3A). To test the robustness of these trends to sampling biases, we subdivided the Gulf of Maine into east, west, and coastal sections, divided at 68.5 °W and tested the same phenology metrics for trends. Of the 30 significance tests, 10 were significant at the 0.05 level, 5 were significant at the 0.01 level, and all significant trends were positive for both spring and autumn metrics. We also tested for trends over smaller sliding windows and found that time series of at least 40 years were necessary for significant trends to emerge. Oceanographic variables had statistically significant trends as well, including increases in temperature and stratification and decreases in nitrate+nitrite and phosphate through much of the year (Fig. 3B).

### Associations

Correlations between phenology metrics and climate indices were generally weak, with only a few correlations at *p* < 0.05. Spring and autumn *τ*_*t*_ correlated positively with the AMO, and autumn *τ*_*t*_ correlated negatively with the NAO (*r*^2^ = 0.17, 0.16, 0.17, respectively). These AMO correlations were also significant at lags of up to three years, with correlations peaking for spring *τ*_*t*_ at a three year lag (*r*^2^ = 0.19), and for autumn *τ*_*t*_ at a two year lag (*r*^2^ = 0.24). Coherence analysis (File S2) also showed weak associations, with *r*^2^ typically below 0.2. The notable exceptions were spring phenology metrics and the AO, at ∼3 year periods, and various metrics and the NAO, at both short and long periods, where *r*^2^ increases to 0.3–0.4.

Correlations between phenology metrics and ocean physics and chemistry showed a moderate signal with some consistent patterns (Table 1). For spring metrics, the strongest signal was a correlation between stratification and earlier bloom timing. Additionally, high February nutrients correlated with a later bloom, and high June nutrients correlated with an earlier bloom. For autumn metrics there was a consistent pattern across all months of high temperature and salinity correlating with earlier bloom initiation. There was a very strong correlation between October silicate and earlier bloom timing, but these correlations consisted of only four data points.

**Table 1:** Correlation coefficients between the phenology metrics and the monthly temperature (T), salinity (S), and stratification (Δ) values, and the seasonal anomalies of nitrate+nitrite (N), silicate (Si), and phosphate (P). Each column represents the month or season over which the physical variable or nutrient anomaly was averaged. Numbers in parentheses indicate correlation coefficients (*r*, *p*). Only correlations with *p* < 0.05 shown.

**SPRING METRICS**					
J	F	M	A	M	J
**Physical variables**					
	Δ, *τ*_*t*_(−0.93, < 0.01)				T, *μ*_*g*_(−0.41, 0.03)
**Chemical variables**					
	N, *μ*_*m*_(0.61, 0.05)				N, *μ*_*g*_(−0.53, 0.04)
	N, *μ*_*c*_(0.66, 0.03)				Si, *μ*_*g*_(−0.55, 0.03)
**AUTUMN METRICS**					
**J**	**A**	**S**	**O**	**N**	**D**
**Physical variables**					
T, *τ*_*d*_(−0.61, 0.02)	T, *τ*_*d*_(−0.68, < 0.01)	T, *τ*_*d*_(−0.49, < 0.01)	T, *τ*_*d*_(−0.40, < 0.01)	T, *τ*_*d*_(−0.50, < 0.01)	S, *τ*_*t*_(−0.84, 0.03)
T, *τ*_*t*_, (−0.55, 0.01)	S, *τ*_*d*_(−0.66, < 0.01)	S, *τ*_*d*_(−0.71, 0.03)	S, *τ*_*d*_(−0.64, 0.05)	S, *τ*_*d*_(−0.70, 0.01)	S, *τ*_*d*_(−0.64, 0.01)
Δ, *τ*_*d*_(−0.48, < 0.01)					S, *μ*_*g*_(−0.45, 0.02)
Δ, *τ*_*t*_(−0.55, 0.03)					
**Chemical variables**					
	N, *μ*_*g*_(−0.51, 0.04)		Si, *μ*_*g*_(−0.98, 0.04)		
			Si, *μ*_*m*_(−0.96, 0.02)		
			Si, *τ*_*d*_(−1.00, < 0.01)		

### Grazer analysis

**Figure 4:**
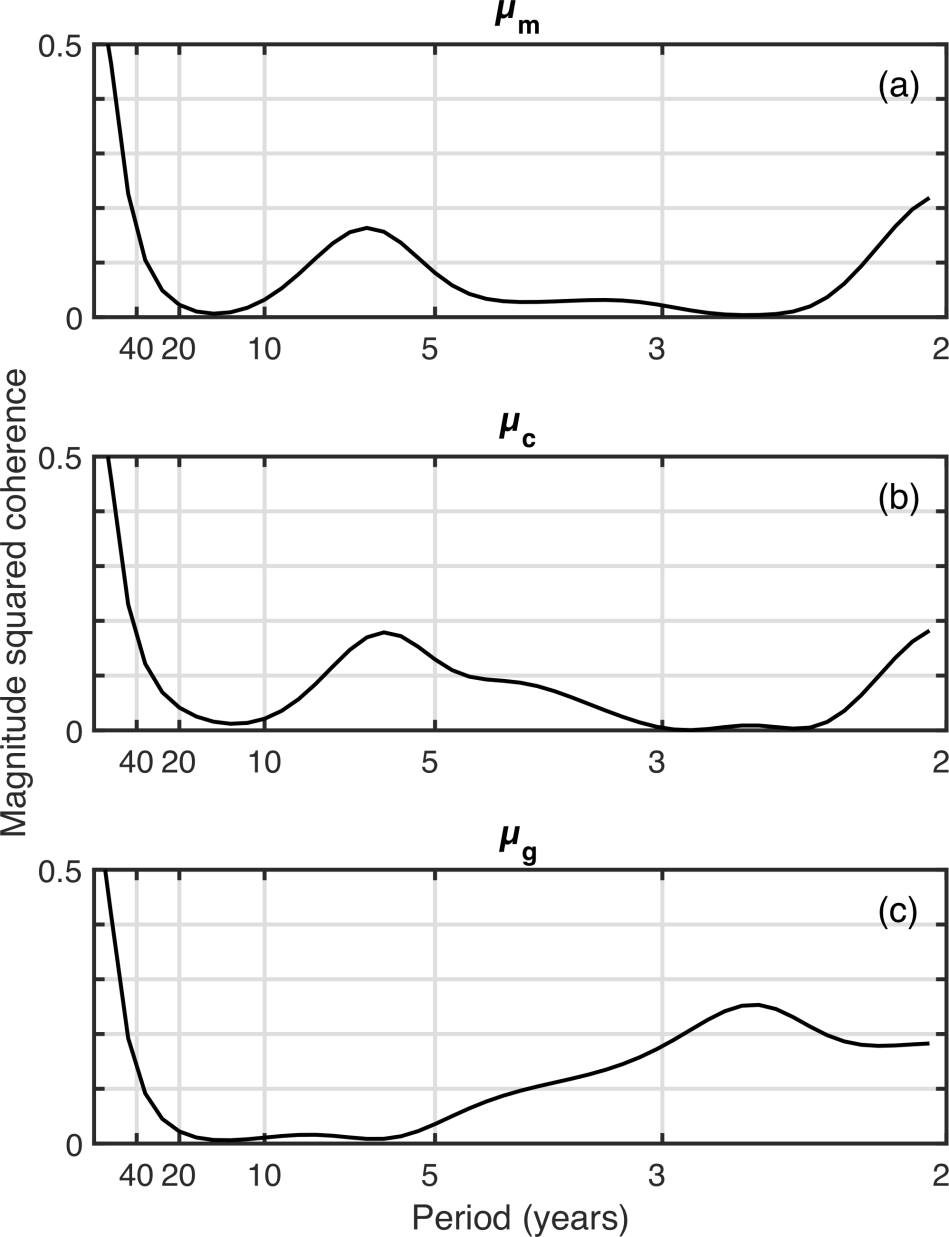
Coherence analysis of three spring phenology metric time series against the time series of late-stage *C. finmarchicus* abundance.

The *C. finmarchicus* index correlated significantly with *μ*_*g*_ (*r*^2^ = 0.17,  *p* = 0 .01). The western Gulf of Maine *C. finmarchicus* population is supplied by coastal waters, so we compared the phenology metrics to the *C. finmarchicus* in the eastern Gulf of Maine (> − 68^∘^) and found stronger and more consistent correlations: *μ*_*m*_ (*r*^2^ = 0.17,  *p* = 0 .01), *μ*_*c*_ (*r*^2^ = 0.24,  *p* = 0 .003), *μ*_*g*_ (*r*^2^ = 0.26,  *p* = 0 .001). Coherence analysis (Fig. 4) showed that these correlations were largely driven by the long-term secular trend. There was a dip in coherence for periods of approximately 10–40 years. There was also an increase in coherence for periods of <10 years to *r*^2^ ≈ 0.2, depending on the metric.

## Discussion

**Figure 5:**
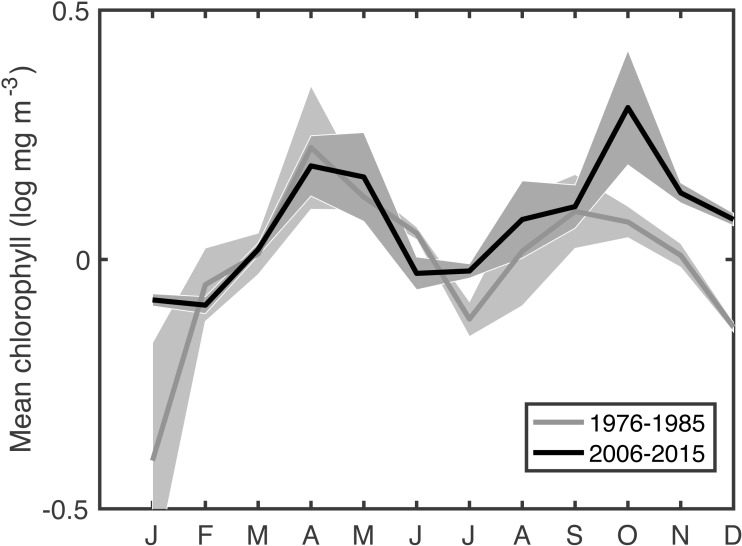
Comparison of mean seasonal cycle of chlorophyll between the most recent decade and an earlier period. Shaded area shows the variance.

This extended time series of in situ chlorophyll measurements in the Gulf of Maine gives a climate-scale perspective on changing phenology that is not available in the comparatively recent satellite records. Climate-scale changes in the timings of blooms are clear, as are associations between bloom phenology and changing physical conditions as well as changing *C. finmarchicus* abundance. The most surprising result is that spring bloom timing, primarily using measures of the bloom center, have gotten later at a rate of ∼1–9 days per decade. This shift is apparent when comparing the recent seasonal cycle to that of a few decades ago (Fig. 5). Typically, as the climate warms, summer lengthens, shifting spring events earlier and autumn events later. The shift toward later autumn blooms is consistent with this expectation, but the shift toward later spring blooms is unexpected in this context. Climate indices have been cited to explain recent temperature and ecosystem changes in the Gulf of Maine (Pershing et al., 2015). However, our analysis did not reveal any compelling relationship with the NAO, AMO, GSI, AOO, or AO–the climate oscillations known to be important in the Gulf of Maine—either via direct correlation, lagged correlation, or coherence analysis.

Relationships between bloom phenology and water properties were somewhat more promising. A later spring bloom was associated with lower stratification, higher nutrients near the beginning of the bloom, and lower nutrients near the end of the bloom. Causality is difficult to assign regarding nutrients. Because an earlier spring bloom correlates with decreased February nutrients, and a later spring bloom with decreased June nutrients, it is likely that the bloom is driving the nutrient dynamics through drawdown (or lack thereof), rather than the reverse. The autumn bloom was more tightly associated with changes in physics. Low temperature and salinity are both associated with increased transport of Arctic-origin water via the Labrador Current, which altered autumn bloom patterns during the 1990s (MERCINA Working Group, 2012). The pattern seen here suggests similar physical forcing might have a more consistent long-term effect specifically on the timing of autumn bloom initiation.

It is worth noting the secular trends in conditions, as well as correlations, focus on high-frequency variability and can overlook climate-scale changes. Spring conditions have shifted towards higher stratification and lower nutrients. The timing shift of the spring bloom is consistent with short-term shifts in the three Gulf of Maine zones described by (Ji et al., 2007); they hypothesize that higher stratification causes earlier blooms upstream on the Scotian Shelf, leading to depleted nutrients and later blooms by the time the water mass reaches the central Gulf of Maine. This hypothesis is consistent with our long-term observations and may provide a better explanation for the shift toward later spring blooms.

The positive association between spring bloom timing and late spring *C. finmarchicus* abundance is notable. If later timing of the center of the bloom is an indication of an extended bloom, then *C. finmarchicus* emerging from diapause could have a longer window for reproduction, and the first generation cohort would be more abundant than in a typical year. This offers one possible explanation for why the species has persisted in the western Gulf of Maine despite rapid warming and temperatures that should be detrimental to the population (Runge et al., 2014), but would require a consistent upstream supply of late-stage individuals. This shift also runs counter to the expectation of a phenology “mismatch” as conditions change (Edwards & Richardson, 2004).

While this chlorophyll data set gives us new insights into climate-scale phenology changes, there are some caveats to bear in mind. First, chlorophyll is a proxy for phytoplankton. When the oceanography changes, adaptation or replacement of species can occur, and chlorophyll measurements might not capture this. As a means for detecting the major phenological events (i.e., blooms), chlorophyll is probably effective, but this caveat should be kept in mind. Second, the dataset includes measurements that use different methodologies. Measuring chlorophyll “*is endlessly complicated by diverse methods of collection and analysis, each with its own virtues but only imperfectly comparable with other methods*” (Riley, 1949). Again, the fact that we use relative changes to compute phenology metrics should hedge this caveat, but it should be considered as the data are used in the future.

## Conclusion

The shift of both spring and autumn bloom timing toward later dates is surprising. The high range in rate estimates is indicative of the difficulty of quantifying bloom phenology, so the emphasis should be on the consistent directionality across metrics rather than any specific rate. Finally, while the analysis here is suggestive of possible drivers of the shift in bloom timing, a mechanistic explanation for bloom initiation has been an elusive goal in oceanography. As Evans & Parslow (1985) wrote: “*Although it seems intuitively reasonable that a sudden effect should have a sudden cause, ecological systems need not behave intuitively. ...[S]pring blooms can occur without any sudden changes in driving variables.*” The Gulf of Maine, with its unprecedented rapid changes (Pershing et al., 2015) is often seen as a potential bellwether for other marine systems. The apparent climate-scale shift toward later spring blooms underscores the point that ecosystems do not always change as expected.

##  Supplemental Information

10.7717/peerj.6735/supp-1 Dataset S1 Aggregate dataset of chlorophyll measurements from the inner Gulf of Maine used in this study

10.7717/peerj.6735/supp-2 Supplemental Information 1 Results of coherence analysis not shown in manuscript

## Additional Information and Declarations

### Data Availability

The following information was supplied regarding data availability: The raw data are available as Dataset S1.

## References

[ref-1] Balch W, Huntington T, Aiken G, Drapeau D, Bowler B, Lubelczyk L, Butler K (2016). Toward a quantitative and empirical dissolved organic carbon budget for the Gulf of Maine, a semienclosed shelf sea. Global Biogeochemical Cycles.

[ref-2] Behrenfeld MJ, Boss ES (2014). Resurrecting the ecological underpinnings of ocean plankton blooms. Annual Review of Marine Science.

[ref-3] Benesty J, Chen J, Huang Y (2005). A generalized MVDR spectrum. IEEE Signal Processing Letters.

[ref-4] Bigelow H (1924a). Plankton of the offshore waters of the Gulf of Maine. Bulletin of the Bureau of Fisheries.

[ref-5] Bigelow HB (1924b). Physical oceanography of the gulf of maine. Bulletin of the Bureau of Fisheries.

[ref-6] Boyce DG, Lewis M, Worm B (2012). Integrating global chlorophyll data from 1890 to 2010. Limnology and Oceanography: Methods.

[ref-7] Boyer TP, Antonov JI, Baranova OK, Coleman C, Garcia HE, Grodsky A, Johnson DR, Locarnini RA, Mishonov AV, O’Brien TD, Paver CR, Reagan JR, Seidov D, Smolyar IV, Zweng MM, Levitus S, Mishonov A (2013). World ocean database 2013, NOAA Atlas NESDIS 72.

[ref-8] Brody SR, Lozier MS, Dunne JP (2013). A comparison of methods to determine phytoplankton bloom initiation. Journal of Geophysical Research: Oceans.

[ref-9] Cushing D (1969). The regularity of the spawning season of some fishes. ICES Journal of Marine Science.

[ref-10] Drinkwater K, Gilbert D (2004). Hydrographic variability in the waters of the Gulf of St. Lawrence, the Scotian Shelf and the eastern Gulf of Maine (NAFO subarea 4) during 1991–2000. Journal of Northwest Atlantic Fishery Science.

[ref-11] Edwards M, Richardson AJ (2004). Impact of climate change on marine pelagic phenology and trophic mismatch. Nature.

[ref-12] Evans GT, Parslow JS (1985). A model of annual plankton cycles. Biological Oceanography.

[ref-13] Foukal NP, Thomas AC (2014). Biogeography and phenology of satellite-measured phytoplankton seasonality in the California Current. Deep Sea Research Part I: Oceanographic Research Papers.

[ref-14] Golet WJ, Record NR, Lehuta S, Lutcavage M, Galuardi B, Cooper AB, Pershing AJ (2015). The paradox of the pelagics: why bluefin tuna can go hungry in a sea of plenty. Marine Ecology Progress Series.

[ref-15] Gregory D (2004). Climate: a database of temperature and salinity observations for the northwest Atlantic. Fisheries & Oceans Canada, Science, Canadian Science Advisory Secretariat.

[ref-16] Henson SA, Sarmiento JL, Dunne JP, Bopp L, Lima ID, Doney SC, John JG, Beaulieu C (2010). Detection of anthropogenic climate change in satellite records of ocean chlorophyll and productivity. Biogeosciences.

[ref-17] Ji R, Davis CS, Chen C, Townsend DW, Mountain DG, Beardsley RC (2007). Influence of ocean freshening on shelf phytoplankton dynamics. Geophysical Research Letters.

[ref-18] Ji R, Edwards M, Mackas DL, Runge JA, Thomas AC (2010). Marine plankton phenology and life history in a changing climate: current research and future directions. Journal of Plankton Research.

[ref-19] Mahadevan A, D’Asaro E, Lee C, Perry MJ (2012). Eddy-driven stratification initiates North Atlantic spring phytoplankton blooms. Science.

[ref-20] McShane BB, Gal D (2017). Statistical significance and the dichotomization of evidence. Journal of the American Statistical Association.

[ref-21] MERCINA Working Group (2012). Recent Arctic climate change and its remote forcing of Northwest Atlantic shelf ecosystems. Oceanography.

[ref-22] Pershing AJ, Alexander MA, Hernandez CM, Kerr LA, Le Bris A, Mills KE, Nye JA, Record NR, Scannell HA, Scott JD (2015). Slow adaptation in the face of rapid warming leads to collapse of the Gulf of Maine cod fishery. Science.

[ref-23] Pershing AJ, Record NR, Monger BC, Mayo CA, Brown MW, Cole TV, Kenney RD, Pendleton DE, Woodard LA (2009). Model-based estimates of right whale habitat use in the Gulf of Maine. Marine Ecology Progress Series.

[ref-24] Raitsos DE, Reid PC, Lavender SJ, Edwards M, Richardson AJ (2005). Extending the SeaWIFS chlorophyll data set back 50 years in the northeast Atlantic. Geophysical Research Letters.

[ref-25] Rebuck N, Townsend D (2014). A climatology and time series for dissolved nitrate in the Gulf of Maine region. Deep Sea Research Part II: Topical Studies in Oceanography.

[ref-26] Riley GA (1949). Quantitative ecology of the plankton of the western North Atlantic. Bulletin of the Bingham Oceanographic Collection.

[ref-27] Runge JA, Ji R, Thompson CR, Record NR, Chen C, Vandemark DC, Salisbury JE, Maps F (2014). Persistence of Calanus finmarchicus in the western Gulf of Maine during recent extreme warming. Journal of Plankton Research.

[ref-28] Smith PC, Pettigrew NR, Yeats P, Townsend DW, Han G (2012). Regime shift in the Gulf of Maine. American Fisheries Society Symposium.

[ref-29] Sverdrup H (1953). On vernal blooming of phytoplankton. Journal du Conseil/Conseil Permanent International pour l’Exploration de la Mer.

[ref-30] Taylor JR, Ferrari R (2011). Shutdown of turbulent convection as a new criterion for the onset of spring phytoplankton blooms. Limnology and Oceanography.

[ref-31] Thomas AC, Pershing AJ, Friedland KD, Nye JA, Mills KE, Alexander MA, Record NR, Weatherbee R, Henderson ME (2017). Seasonal trends and phenology shifts in sea surface temperature on the North American northeastern continental shelf. Elementa Science of the Anthropocene.

[ref-32] Thomas AC, Townsend DW, Weatherbee R (2003). Satellite-measured phytoplankton variability in the Gulf of Maine. Continental Shelf Research.

[ref-33] Wernand MR, Van der Woerd HJ, Gieskes WW (2013). Trends in ocean colour and chlorophyll concentration from 1889 to 2000, worldwide. PLOS ONE.

[ref-34] Zarubin M, Lindemann Y, Genin A (2017). The dispersion-confinement mechanism: phytoplankton dynamics and the spring bloom in a deeply-mixing subtropical sea. Progress in Oceanography.

