# Supplementary material for: Century-scale changes in phytoplankton phenology in the Gulf of Maine
Source: PeerJ. 2019 May 2;7:e6735. doi: 10.7717/peerj.6735 (PMC6500720; doi:10.7717/peerj.6735)
Supplement: Supplemental Information 1 [file peerj-07-6735-s002.pdf]

Century-scale changes in phytoplankton phenology in the Gulf of Maine

N. R. Record, W. M. Balch, K Stamieszkin
Bigelow Laboratory for Ocean Sciences, East Boothbay ME, USA

Contents

The output of the coherence analysis, comparing phenology index time series with climate indices, as described in the methods, is shown here.

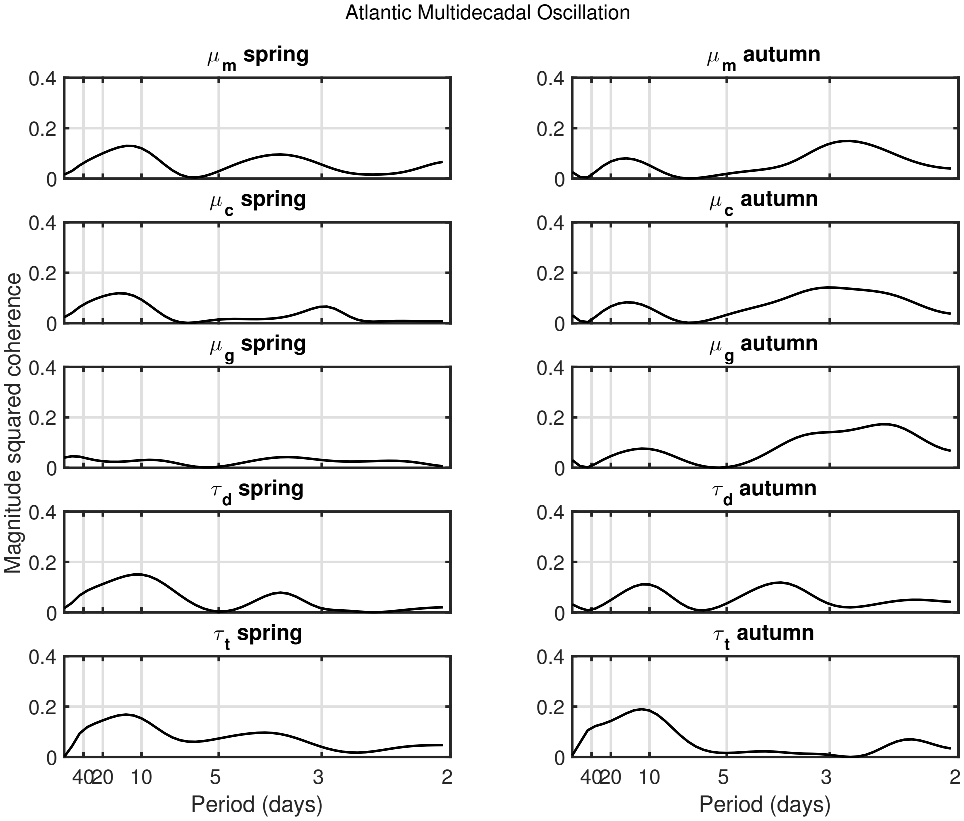

Figure 1: Coherence analysis of phenology metrics and the Atlantic Multidecadal Oscillation.

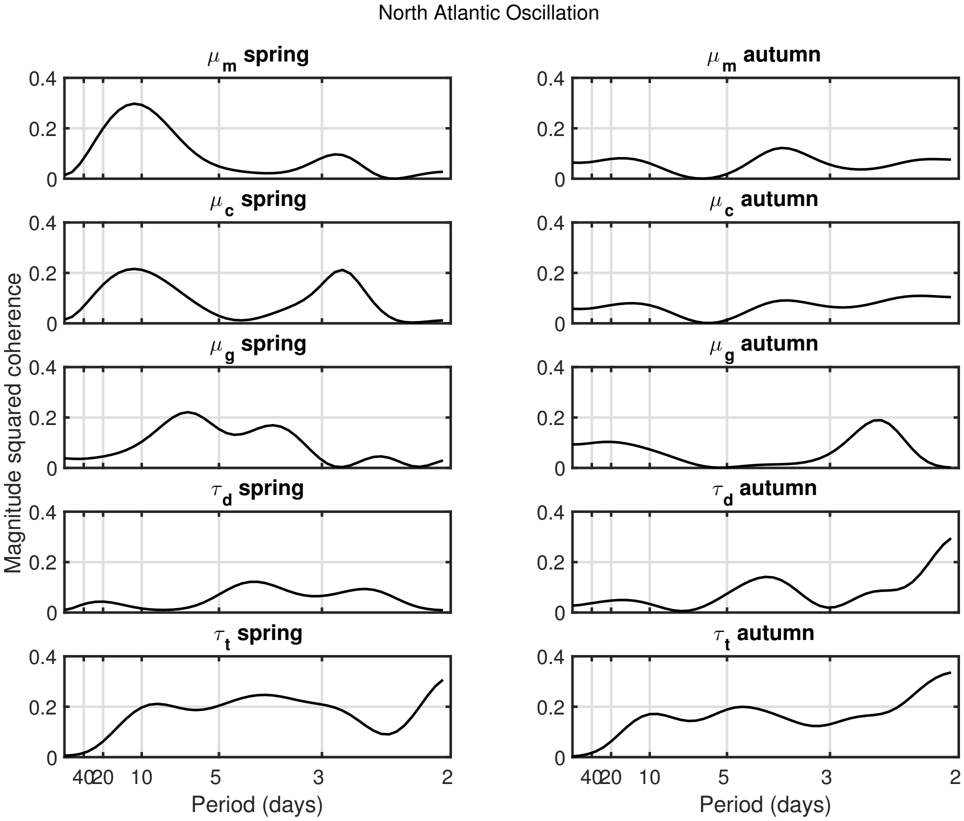

Figure 2: Coherence analysis of phenology metrics and the North Atlantic Oscillation.

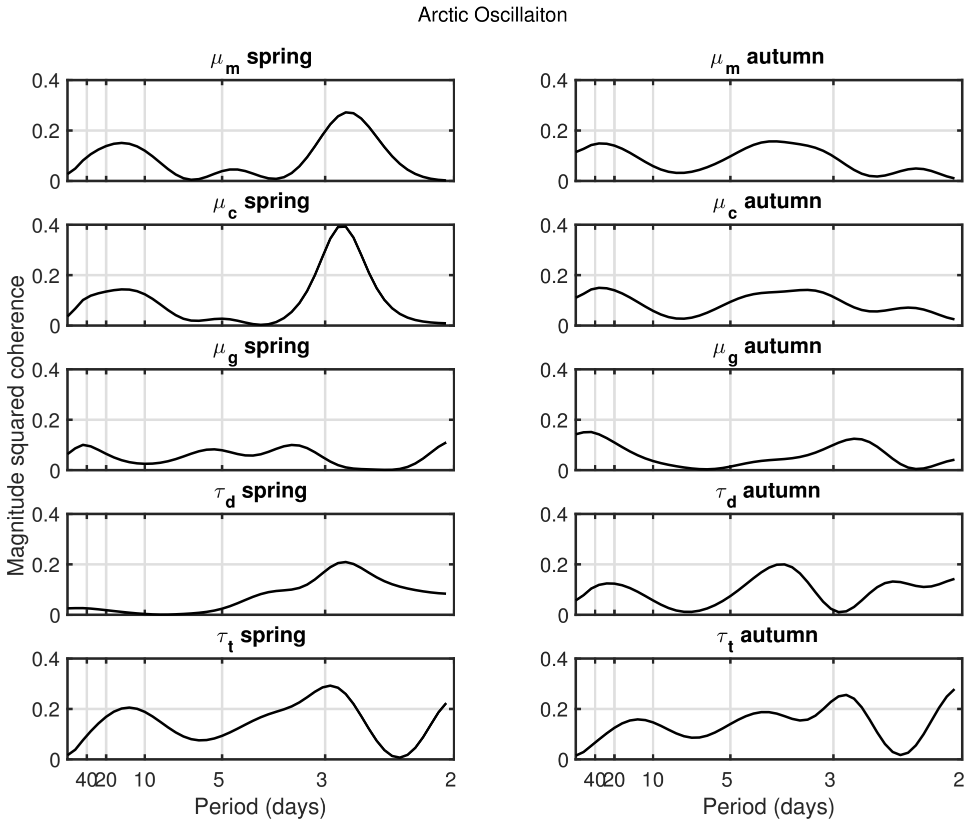

Figure 3: Coherence analysis of phenology metrics and the Arctic Oscillation.

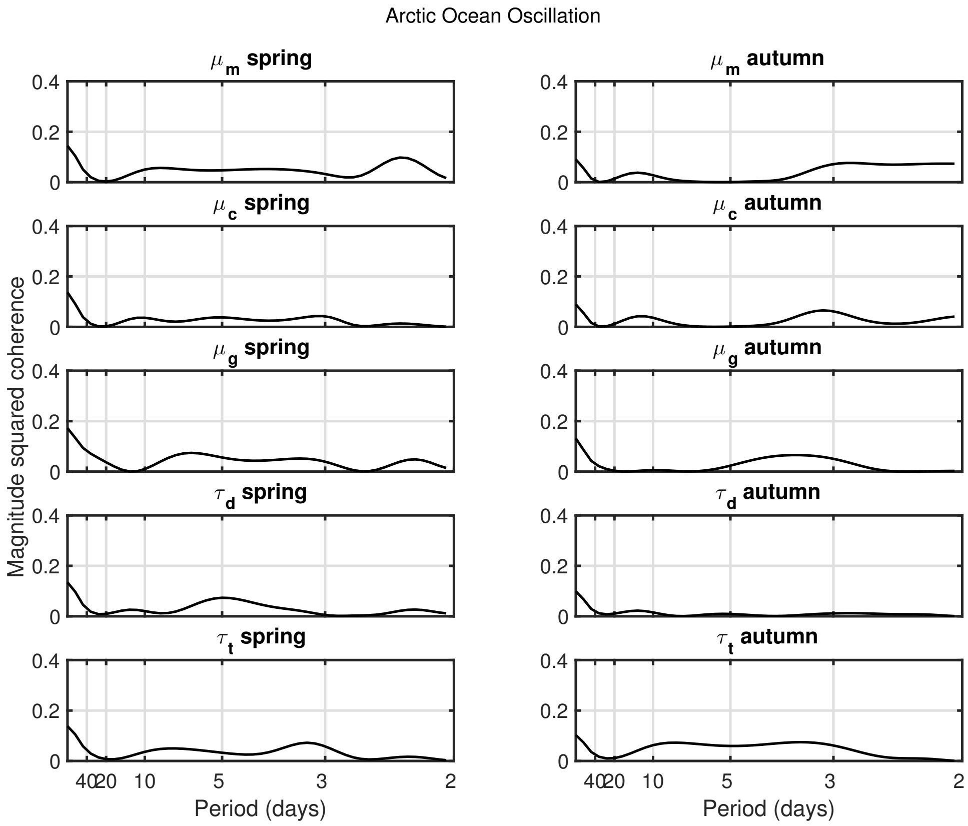

Figure 4: Coherence analysis of phenology metrics and the Arctic Ocean Oscillation.

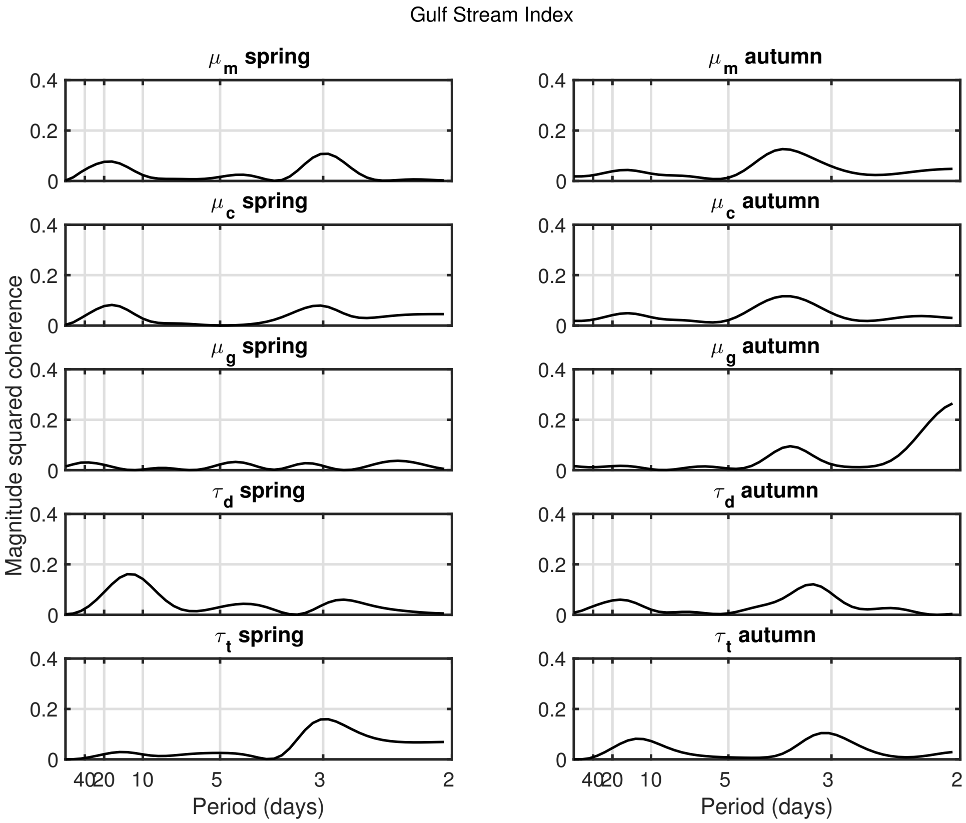

Figure 5: Coherence analysis of phenology metrics and the Gulf Stream Index.
